# Mediators of parental educational differences in the intake of carbonated sugar-sweetened soft drinks among adolescents, and the moderating role of neighbourhood income

**DOI:** 10.1186/s12937-023-00872-7

**Published:** 2023-09-11

**Authors:** Teferi Mekonnen, Eleni Papadopoulou, Nanna Lien, Lene F. Andersen, Maria Gabriela Matias Pinho, Hanne Hennig Havdal, Oddbjørn Klomsten Andersen, Mekdes K. Gebremariam

**Affiliations:** 1 Department of Nutrition, Institute of Basic Medical Sciences, Faculty of Medicine, University of Oslo, Oslo, Norway; 2 Division of Health Service, Global Health Cluster, Norwegian Institute of Public Health, Oslo, Norway; 3 Department Environmental Sciences, Copernicus Institute of Sustainable Development, Utrecht University, Utrecht, the Netherlands; 4 Department of Sports Medicine, Norwegian School of Sport Sciences, Oslo, Norway; 5 Department of Community Medicine and Global Health, Institute of Health and Society, Faculty of Medicine, University of Oslo, Oslo, Norway

**Keywords:** Adolescents, Sugar-sweetened, Soft drink, Mediators, Moderated mediation

## Abstract

**Background:**

Existing evidence suggests that the intake of sugar-sweetened beverages (SSB) among adolescents remains a public health concern and that socioeconomic differences in intake exist. Tackling these challenges requires identifying the factors associated with SSB intake and the mediators of socioeconomic differences in SSB intake among adolescents. Thus, this study aimed to explore (i) factors at different levels of the ecological model associated with the intake of carbonated soft drinks with added sugar (hereafter called soft drinks), (ii) mediators of the association between parental education and the intake of soft drinks(iii) whether neighbourhood income moderates the indirect effect of parental education on adolescents’ soft drink intake through potential mediators.

**Methods:**

Data from 826 7^th^ graders in Oslo, Norway, who participated in the TACKLE cross-sectional study conducted in 2020 were used. The association between factors at the individual, interpersonal and neighbourhood food environment levels and the intake of soft drinks among adolescents was assessed, as well as the mediating roles of these factors for the differences in intake by parental education, using multiple logistic regression and mediation analysis, respectively. Moderated mediation analyses were used to explore whether an indirect effect of parental education on adolescents' soft drink intake through potential mediators varies across neighbourhood income areas.

**Results:**

Higher perceived accessibility of SSB at home, increased parental modelling for SSB intake, and increased frequency of food/drink purchased from the neighbourhood store were associated with a higher intake of soft drinks among adolescents and mediated the differences in intake by parental education. Neighbourhood food environment factors were neither statistically significantly associated with adolescents’ higher intake of soft drinks nor explained the differences in intake by parental education. Moderated mediation analysis showed that the mediating effect of perceived accessibility of SSB at home on the association between parental education and adolescent soft drink intake was stronger among those living in low neighbourhood income.

**Conclusions:**

Our study identified modifiable factors at the intrapersonal level (perceived accessibility of SSB at home and frequency of food/drink purchased from neighbourhood shops) and interpersonal levels (parental modelling for SSB intake) associated with a higher intake of soft drinks among adolescents and mediated the differences in the intake by parental education. The modifiable factors identified in this study could be targeted in public health initiatives among adolescents aimed at reducing the intake of soft drinks and the related differences by parental education.

## Background

The intake of sugar-sweetened beverages (hereafter called SSB)is associated with a higher risk of overweight/obesity [1], type 2 diabetes [2], hypertension [3] and a higher burden of SSB-attributable disability and death [4]. Next to water, SSB are the most consumed beverages among European adolescents [5]. Studies from the European region indicate a trend towards reduced SSB intake among adolescents [6–10]. However socioeconomic differences persist with existing evidence showing that adolescents with a lower socioeconomic position (SEP) have a higher SSB intake than their counterparts with a higher SEP [6, 7, 11, 12].

Adolescence is an important life stage that provides an opportunity for behaviours to be shaped positively; it is thus a critical life period to target in order to combat socioeconomic differences in SSB intake. Tackling socioeconomic differences in SSB intake among adolescents needs a better understanding of factors contributing to these differences.

According to the ecological model of health behaviours, adolescent dietary behaviours, including SSB intake, could be influenced by multilevel interacting factors at the individual, interpersonal/social and broader environmental levels [13]. Various factors at individual, interpersonal/social and broader levels including the neighbourhood environment have been linked with SSB intake among adolescents. Individual-level factors such as preference for soft drinks, screen time/TV viewing and snack intake were associated with higher SSB intake [14, 15]. The association between knowledge, outcome expectations and self-efficacy with SSB intake were on the other hand reported to be inconsistent [14, 15]. At the interpersonal /social level, accessibility, home availability, and peer influence were associated with higher SSB intake [14–16]. Parental modelling for healthy eating and parental rules were associated with a lower intake of SSB [16, 17]. At the broader environmental levels, studies have shown that the availability of SSB in the neighbourhood, and the availability or accessibility of food outlets in the neighbourhood were positively associated with SSB intake among adolescents [14, 17, 18]. Policies that decrease exposure to SSB and ban promotions in schools were associated with a lower SSB intake [19]. For the neighbourhood food environment, inconsistent associations have been found, depending on whether objective or subjective measures are used [18, 20].

Evidence also shows that these determinants can vary by SEP [21] and thus act as mediators of socioeconomic differences in dietary behaviours. In this regard, studies found that perceived rules, perceived accessibility at home, preferences, attitudes, home availability, and parental modelling mediated socioeconomic differences in SSB intake among adolescents [22–25]. These studies have mainly investigated mediators of socioeconomic differences in adolescent SSB intake at the individual and interpersonal levels [22–25]. However, factors at broader levels, such as the neighbourhood food environment, have been less studied. Socioeconomic differences in neighbourhood accessibility of supermarkets/grocery stores [26], and fast-food outlets/convenience stores [26, 27] have been observed. The same is true for the availability of food outlets [28]. However, there is limited evidence regarding the mediating roles of neighbourhood food environmental factors in the association between socioeconomic position and dietary behaviours among youth, including SSB intake [29]. Thus, more studies that consider neighbourhood food environmental factors (i.e. both perceived and objective measures) while exploring the factors influencing SSB intake among adolescents and the mediators of socioeconomic differences in SSB intake are needed. In addition, studies showed differences in dietary behaviours by neighbourhood income even after controlling for individual-level SEP [30, 31]. The associations between individual-level SEP (e.g. parental education) and dietary behaviours, including SSB intake, may thus potentially be moderated by neighbourhood income. However, to the authors' knowledge, studies exploring whether the indirect effect of parental education on adolescents’ SSB intake through the potential mediators can be moderated by neighbourhood-income are lacking.

Therefore, this study aimed to explore (i) factors including broader neighbourhood food environmental factors associated with the intake of carbonated soft drinks with added sugar (hereafter called soft drinks) among adolescents, (ii) mediators of the association between parental education and soft drink intake among adolescents, and (iii) whether neighbourhood income moderates the indirect effect of parental education on adolescents’ soft drink intake through potential mediators.

## Methods

### Design and sample

This study utilized data from the Tackling Socioeconomic Differences in Weight Development among Youth (TACKLE) study. The TACKLE study was a school-based cross-sectional study that included participants enrolled in the 7^th^ grade (i.e., final year of primary school in Norway). A total of 94 primary schools in Oslo were invited, and 28 schools participated in the study. Prior to the invitation, schools were assessed for eligibility, and special schools and those with few students in the 7^th^ grade were excluded. A total of 1540 students were invited to participate. Written informed consent from a parent or legal guardian was obtained for 939 (63%) of these students. Of the 897 students (58%) who participated in the TACKLE study, 826 (53.6%) adolescents had data on soft drink intake and parental level of education.

Data collection was performed at two different time points due to Covid-19. From February–March 2020, 11 schools participated, and from September–November 2020, 17 schools participated.

### Data collection and procedure

Data were collected using an internet-based questionnaire filled in by the students in their classroom or computer room. Research personnel from the University of Oslo and teachers from the respective schools were present to answer questions, resolve technical issues and ensure that the students replied independently from each other. A pilot test and test–retest study were conducted before the data collection to ensure the reliability and validity of the questionnaire.

### Outcome variable

In this study, soft drink intake was defined as the intake of carbonated soft drinks with added sugar. Adolescents' intake of soft drinks was assessed by asking about the frequency of soft drink intake during weekday and weekend days using a food frequency questionnaire modified from the ENERGY-child questionnaire [32]. Adolescents’ weekday intake of soft drinks was assessed by asking about the frequency of soft drink intake from Monday through Friday with response options ranging from never/rarely to 5 days and the amount consumed in glasses (0.25 L (L)), cans (0.33L) or bottles (0.5L) with a response option ranging from none to five or more glasses/cans /bottles. Intake of soft drinks during the weekend was assessed by asking the amount consumed in glasses (0.25 L (L)), cans (0.33L) or bottles (0.5L) during the weekend days with response options ranging from none to five or more glasses/cans /bottles.

After computing the weekday and weekend intake of soft drinks by combining frequency and amount, the weekly intake of soft drinks was calculated by summing the intakes during weekday and weekend intake. Given the weekly intake of soft drinks was not normally distributed, the median intake of soft drinks was used to generate a binary soft drinks intake outcome variable (i.e. 1L/week). Accordingly, adolescent soft drink intake was recoded as " lower intake of soft drinks" and "higher intake of soft drinks " for those having a median weekly intake of soft drinks ≤ 1L/week and > 1L /week, respectively. The test–retest reliability for the weekly intake measure was good (ICC = 0.65).

### Exposure variable: parental level of education

The adolescents' socioeconomic background was assessed based on parental level of education from a paper-based questionnaire by asking the level of education in six categories (i.e. no education/has not completed primary school to completed university/college education (> 4 years)) filled in by the parents as part of the consent procedure. Then, the parental level of education variable with three categories of "low", "medium" and "high" education was created according to the years of education completed: up to vocational school, completed university/college up to four years and completed university/college for more than four years, respectively.

### Potential individual and interpersonal correlates

Self-efficacy for healthy eating was adopted and modified from Dewar et al. [33] and demonstrated good test–retest reliability. Perceived maternal and paternal norms for healthy eating were modified from Baker et al. [34] and it showed moderate test–retest reliability. Parental rules for SSB intake and perceived accessibility of SSB at home were adopted and modified from Bjelland et al. [35] and good test–retest reliability was found. Perceived maternal and paternal modelling was modified from De Bourdeaudhuij et al. [36, 37] and showed excellent test–retest reliability. Food purchasing behaviour was modified from Gebremariam et al. [38] and showed good test–retest reliability. The details about how the correlates at the individual and interpersonal were assessed and the test–retest reliability estimates are presented in Table 1.

**Table 1:** Example of items, source and test–retest reliability test results of potential determinants included in the study

Variables with example items asked	Response categories	ICC and/ or percentage agreement**
Self-efficacy*; “*e.g****.**** I find it easy to choose a healthy snack when I eat in-between meals (e.g. fruit or reduced-fat yoghurt) *[31]*”*	5 point likert-type scale ranging from 1(strongly disagree) to 5(strongly agree)	0.61
Maternal and paternal norms*; *“My mother/father thinks I should eat healthily; My mother/father is a healthy eater *[32]*.”*	5 point likert scale ranging from 1(strongly disagree) to 5(strongly agree)	0.4- 0.7 for maternal norms; 0.35–0.36 (70–80%) for paternal norms
Parental rules regarding adolescent SSB intake; *“my parents have clear rules for how much sugar-sweetened beverages (e.g. fizzy drinks, fruit squash, cordials *etc.) I can drink [33].”	Answer categories ranging from 1(not at all) to 5(very)	0.67
Perceived accessibility of SSB at home*; *“e.g. at home, we usually have soft drinks for dinner at the weekend *[33]*”*	5 point likert-type scale ranging from 1(strongly disagree) to 5(strongly agree)	0.67
parental modelling for SSB intake*; *“ my mother/mother drinks fizzy drinks with added sugar several times a week *[34]*. “*	5 point likert-type scale ranging from 1(strongly disagree) to 5(strongly agree)	0.66-maternal modelling 0.81-paternal modelling
Food purchasing behaviours;*” How often do you usually purchase food/drinks in shops (grocery shop, kiosk, gas station) close to your school or your neighbourhood?* [35]*”*	A categorical response with 7 response categories (i.e. never, every other week, once a week, twice a week, 3 times a week, 4–5 times a week, 6 times or more)	0.69
Perceived neighbourhood accessibility of SSB; *“ There is a large variety of sugar-sweetened drinks that I like available close to my school/neighbourhood where I purchase food/drink *[35]*.”*	5 point likert-type scale ranging from 1(strongly disagree) to 5(strongly agree)	0.59
Perceived neighbourhood accessibility of food outlets (grocery stores, kiosks/gas stations, fast food places);*”e.g.There are grocery stores (e.g. Kiwi, Rema 1000), within easy walking distance from my home* [36]*.”*	5 point likert-type scale ranging from 1(strongly disagree) to 5(strongly agree)	-
Perceived travel time to the nearest stores;*” e.g.about how long would it take you to walk from your home to the nearest grocery store….*etc*.”*	categorical, with 6 response categories (i.e.1–5 min, 6–10 min, 11–20 min, 21–30 min, 31 min or more, I don’t know	travel time to kiosk/ gas stations(0.53), grocery stores(0.65), fruit and vegetable store(0.64), and fast-food outlets (0.67)
Perceived price of food items;*”e.g. It is cheaper to buy soft drinks or snacks (e.g. biscuits or chips) than buying fruit and vegetables.”*	5 point likert-type scale ranging from 1(strongly disagree) to 5(strongly agree)	0.75

^*^variable with multi-item scale and in that case, the mean score was computed,, **percentage agreement was reported for those with low ICC. *ICC* intraclass correlation

### Perceived measures of neighbourhood food environment

Perceived neighbourhood accessibility of SSB and perceived price of food items were modified from Gebremariam et al. [38] and good test–retest reliability was found. Perceived neighbourhood accessibility of food retailers was modified from Rosenberg et al. [39]. Perceived travel time to the nearest store showed good test–retest reliability. Table 1 presents details of the measures used to assess the perceived neighbourhood food environment, including the test–retest reliability estimates (Table 1).

### Objectively measured neighbourhood food environment

The neighbourhood food environment was measured using ArcGIS Pro 2.6.1(Esri). Participants' addresses were geocoded into ArcGIS. According to a systematic review by Engler-Stringer (2014) on the relationship between food environment and children from 5–18 years old, although great variation exists in chosen buffer sizes, the majority of studies used buffers ranging from 500 to 1000 m [18]. Therefore, given the age of our participants, we defined individual neighbourhoods as a 500-m road network buffer around the participant's home. Historical data on fast food outlets, restaurants and grocery stores were obtained from Prognosesenteret (https://prognosesenteret.no/) and Geodata (https://geodata.no/). The Prognosesenteret and Geodata AS provides data on grocery stores and all types of restaurants, including fast food outlets, hamburger restaurant and pizza restaurant. The following variables were created: “grocery stores”, “fast food outlets” (defined by merging fast food outlets, hamburger restaurants and pizza restaurants) and “all restaurants” (defined as all restaurants excluding the category of “all fast-food outlets”). The locations of all food retailers were verified using Google Street View, an approach previously validated in a Norwegian context [40].

We summarised the number of food retailers (e.g. grocery stores, fast-food outlets and restaurants) within each buffer zone and calculated the density of food retailers. The density of food retailers per neighbourhood area (km^2^) within 500m road-network buffers around the participant’s home address was calculated by dividing the total number of food outlets by neighbourhood area in square kilometres, as defined in other studies [41, 42]. Then, categorical variables representing the density of food retailers which were defined based on the distribution of density measures were generated for the density of restaurants (0, < 3 and ≥ 3 restaurants), the density of grocery stores (0, < 4 and ≥ 4 grocery stores) and the density of fast-food outlets (0, < 5 and ≥ 5 fast-food outlets). In addition, variables which measure the distance (based on the road network buffers) to the closest fast-food outlets, grocery stores and restaurants were generated and used for further analysis.

### Potential moderator

The sub-city district related to the adolescents’ residential address was identified using a document provided by Oslo Municipality [43]. Average mean income by sub-city district was extracted from Statistics Norway. A binary neighbourhood income (low vs. high) was computed using average neighbourhood income (510 000 NOK).

### Covariates

The potential covariates included were sex, age, family structure (lives with both parents vs. other living conditions), data collection period (i.e. pre-corona vs. post-corona lockdown) and ethnicity. Ethnicity was assessed by asking adolescents about their mother’s and father’s country of birth (e.g. what is your mother's/father's country of birth with two response categories (Norway and another country)) and recoded into ethnic Norwegian vs. ethnic minority (both parents born in a country other than Norway) [44].

### Data analyses

Chi-squared test for categorical variables and one-way ANOVA for continuous variables were used to explore parental education differences in adolescents' carbonated soft drinks with added sugar intake, potential correlates, and covariates.

Binary logistic regression analysis was performed to identify factors associated with adolescents' soft drink intake. The variables with *p* < 0.2 in the univariate analyses were included in the final multivariable logistic model. The multivariate model was adjusted for ethnicity, sex, age, family structure, parental education, and data collection period. Odds ratios (OR) with 95% confidence intervals (CI) were generated, and variables with *p*-value ≤ 0.05 were considered statistically significant.

**Fig. 1:**
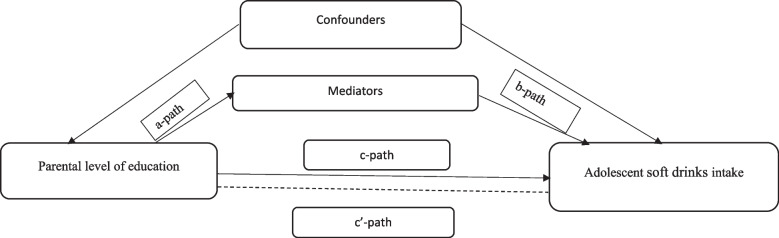
Causal diagram of the association between parental education and adolescents’ soft drinks intake. The a-path represents the association between parental education and the mediators, the b-path represents the association between the mediators and soft drinks intake (adjusted for parental education and the other mediators.), the c’ path represents the association between parental education and soft drink intake (adjusted for the mediators), the c-path represents the unadjusted association between parental education and soft drink intake. Confounders: sex, age, ethnicity, family structure and data collection period

Mediation analyses were performed to identify mediators explaining differences by parental education in adolescents’ soft drink intake. Figure 1 depicts the hypothesised causal relationship between the exposure variable (parental education), potential mediators and the outcome variable (soft drink intake) among adolescents in a multiple mediation model (Fig. 1). In the figure, a-paths represent the association between parental education and the mediators. The b-paths represent the association between the mediator and soft drink intake among adolescents, adjusted for parental education and the other mediators. The c' path represents the association between parental education and soft drink intake, adjusted for the mediators. The c path represents the total effect of parental education on adolescents’ soft drink intake. First, single mediation analyses were performed for both individual, interpersonal and the food environment related factors presented in Table 2 and presumed to lay in the causal pathway between the parental education and adolescents' soft drink intake. Significant mediators in the single mediation analyses were entered into the multiple mediation model and presented in a table. A bootstrap-corrected confidence interval using the SPSS PROCESS macro was used to estimate indirect effects [45].

**Table 2:** Characteristics of adolescents who participated in the TACKLE study by parental education, percentage, mean(95% CI) (*N* = 826) ^a^

**Variables**		**Parental level of education**			***P* value**
		**Low (213, 26.8%)**	**Medium (183, 22.2%)**	**High (430, 52.1%)**	
Sex	Female	24.2	21.0	54.8	0.230
	Male	27.7	23.5	48.8	
Ethnicity	Ethnic Norwegian	12.8	23.4	63.8	**< 0.001**
	Ethnic minority	57.4	19.1	23.5	
Family structure	Lives with both parents	19.9	21.3	58.8	**< 0.001**
	Other living conditions	41.6	25.2	33.2	
Neighbourhood income	High	11.3	21.1	65.6	**< 0.001**
	Low	40.0	23.2	36.8	
Soft drinks intake	Lower intake/week	20.2	18.0	61.8	**< 0.001**
	Higher intake/week	32.3	27.0	40.7	
Age (years)		12.4 (12.3, 12.4)	12.4 (12.3, 12.4)	12.4 (12.4, 12.5)	**0.012**
Self-efficacy		3.2 (3.4, 3.6)	3.7 (3.5, 3.8)	3.7 (3.6, 3.8)	**0.032**
Rules for SSB intake		3.5 (3.3,3.7)	3.6 (3.4, 3.8)	3.7 (3.6, 3.8)	0.171
Perceived accessibility of SSB at home		2.1 (1.2, 2.3)	2.2 (2.1, 2.3)	1.9 (1.8, 1.9)	**< 0.001**
Parental modeling for SSB intake		1.7 (1.6, 1.8)	1.8 (1.6, 1.9)	1.6 (1.5, 1.6)	**0.009**
Perceived price of food item		2.7 (2.5, 2.8)	2.8 (2.6, 3.0)	2.7 (2.6, 2.8)	0.634
Maternal norm		4.5 (4.4, 4.6)	4.6 (4.5, 4.7)	4.7 (4.6, 4.7)	**0.035**
Paternal norm		4.6 (4.5, 4.6)	4.6 (4.5, 4.7)	4.7 (4.7, 4.7)	**0.001**
Perceived accessibility of SSB in the neighborhood stores		3.5 (3.4, 3.7)	3.5 (3.3, 3.7)	3.7 (3.5, 3.8)	0.402
Frequency of food/drink purchase from the neighborhood shops		1.8 (1.6,2.0)	1.2 (1.0, 1.4)	1.0 (0.9, 1.1)	**< 0.001**
Perceived neighborhood accessibility of grocery stores		4.5 (4.4, 4.7)	4.5 (4.38, 4.69)	4.7 (4.6, 4.8)	0.074
Perceived neighborhood accessibility of kiosk		3.8 (3.7, 4.0)	4.0 (3.8, 4.2)	4.0 (3.9, 4.1)	0.275
Perceived neighborhood accessibility of fast-food shops		3.7 (3.5, 3.9)	3.5 (3.3, 3.7)	3.4 (3.3, 3.5)	**0.025**
Travel time to the nearest grocery stores(minutes)		7.1 (6.3, 8.0)	7.1 (6.4, 7.9)	6.5 (6.0,7.0)	0.295
Travel time to the nearest kiosks (minutes)		11.8 (10.5, 13.1)	11.2 (9.9, 12.4)	11.4 (10.6, 12.3)	0.741
Travel time to the nearest fruit and vegetable stores (minutes)		11.2 (10.0, 12.5)	12.5 (11.0, 13.9)	12.5 (11.6, 13.4)	0.229
Travel time to the nearest fast-food shops (minutes)		13.7 (12.5, 15.0)	16.4 (15.0, 17.9)	16.3 (15.4, 17.2)	**0.002**
Distance to the nearest grocery stores (in meters)		454.2 (409.8, 498.6	561.2 (512.0, 610.4)	543.6 (513.1, 574.1)	**0.002**
Distance to the nearest total fast-food outlets (in meters)		675.0 (614.0, 736.1)	759.6 (698.1, 821.0)	741.9 (697.3, 786.6)	0.176
Distance to the nearest restaurants (in meters)		922.7 (747.5, 991.2)	878.2 (786.7, 969.7)	966.7 (895., 1038.2	0.231
Density of grocery stores per neighbourhood area(km^2^) within 500 m network buffers	0 grocery store	22.5	33.3	40.4	**< 0.001**
	< 4 grocery stores	27.0	31.6	25.4	
	≥ 4 grocery stores	50.5	33.3	34.1	
Density of fast-food outlets per neighbourhood area (km^2^) within 500 m network buffers	0 fast-food outlet	46.9	56.9	52.5	**0.008**
	< 5 fast-food outlets	27.5	27.0	33.2	
	≥ 5 fast-food outlets	25.5	16.1	14.3	
Density of restaurants per neighbourhood area (km^2^) within 500 m network buffers	0 restaurant	54.6	56.3	60.0	**0.023**
	< 3 restaurants	19.9	19.5	11.1	
	≥ 3 restaurants	25.5	24.1	27.9	

^a^n varies slightly between variables due to missing data; soft drinks; chi-square test for categorical variables and one-way ANOVA for continuous variables were used; bold values indicate statistically significant values; values are presented as percentages or means (confidence intervals), *SSB* Sugar-sweetened beverages

Moderated mediation analysis using SPSS PROCESS macro was performed to explore whether an indirect effect of parental education on adolescents' soft drink intake through the potential mediators varies by neighbourhood income using a separate moderated mediation analysis model. Bootstrap-corrected confidence intervals were used to test moderation of the indirect effect by the moderator (i.e. neighbourhood income). Evidence of moderation of the indirect effect was declared if the confidence interval of the index of the moderated mediation does not include zero [46]. Moderated mediation effect was explored for the potential mediators considered in this study.

The models were adjusted for potential confounders. School-level clustering was checked, and only 3.8% of the total variation was at the school level. Thus, multilevel analyses were not conducted.

## Results

The mean age of the adolescents included in this study was 12.4 years (SD = 0.4). Of the total respondents, 46.1% had a high soft drink intake per week (> 1 L/week), 71.5% were ethnic Norwegian, 54.6% were females, 22.2% had parents with a medium education, and 25.8% had parents with low education.

Adolescents with lower-educated parents showed lower self-efficacy compared to their counterparts with medium and higher-educated parents (*p* = 0.032). Adolescents with lower and medium-educated parents showed higher perceived accessibility of SSB at home (*p* < 0.001) than adolescents with higher-educated parents. Adolescents with lower and medium-educated parents showed higher parental modelling for SSB intake compared with their counterparts with higher-educated parents (*p* < 0.001). Adolescents with lower and medium-educated parents showed less strict maternal norms (*p* = 0.035) and paternal norms (*p* = 0.001) compared to adolescents with higher-educated parents. Adolescents with lower-educated parents demonstrated a higher frequency of food/drink purchases from the neighbourhood fast-food shops (*p* < 0.001), higher perceived neighbourhood accessibility of fast-food shops (*p* < 0.025), shorter perceived travel time to the nearest fast-food shops (*p* = 0.002) and shorter distance to the nearest grocery stores (*p* = 0.002) compared to adolescents with medium and higher-educated parents. Adolescents with low and medium-educated parents had a lower density of grocery stores (*p* < 0.001) and restaurants (*p* < 0.023), and a higher density of fast-food outlets within 500 m network buffers from their residence compared to adolescents with higher-educated parents (*p* < 0.008) (Table 2).

### Factors associated with adolescents’ soft drink intake

In the univariate analyses, self-efficacy, parental rules for SSB intake, perceived accessibility of SSB at home, parental modelling for SSB intake, maternal and paternal norms, frequency of food/drink purchased from neighbourhood shops, perceived neighbourhood accessibility of kiosk and density of restaurants were significant factors associated with soft drink intake among adolescents (data not shown).

Table 3 shows multivariable logistic regression output for the factors associated with adolescent soft drink intake. Higher perceived accessibility of SSB at home, increased parental modelling for SSB intake, and increased frequency of food/drink purchased from the neighbourhood stores were significantly associated with a high intake of soft drinks among adolescents.

**Table 3:** Results of multivariable logistic regression analysis of potential factors associated with adolescents’ soft drinks intake (low vs. high) (*N* = 826) *

		**Soft drinks intake**
		**OR (95% CI)**
Self-efficacy		0.88 (0.73, 1.06)
Parental rules for SSB intake		0.87 (0.74, 1.02)
Perceived accessibility of SSB at home		1.63 (1.31, 2.03)*
Parental modelling for SSB intake		1.28 (1.03, 1.60)*
Maternal norm		0.87 (0.63,1.20)
Paternal norm		1.01 (0.76,1.35)
Frequency of food/drink purchased from the neighbourhood shops		1.21(1.05, 1.39)*
Perceived neighbourhood accessibility of kiosk		0.90 (0.79, 1.03)
Density of restaurants	0 restaurant	Ref
	< 3 restaurants	1.44 (0.89, 2.34)
	≥ 3 restaurants	1.23 (0.82, 1.85)

The model is adjusted for gender, age, ethnicity, family structure and parental education and data collection period, * indicates associations significant at *p*-value ≤ 0.05, *OR* Odds ratio, *CI* confidence intervals, *SSB* sugar-sweetened beverages

For every one-unit increase in the score of perceived accessibility of SSB at home, the odds of high soft drink intake (vs. low) among adolescents increased by 63% (OR = 1.63). For every one-unit increase in the score of parental modelling for SSB intake, the odds of high (vs. low) intake of soft drink intake among adolescents increased by 28% (OR = 1.28). Similarly, for every one-unit increase in the score for the frequency of food/drink purchased from the neighbourhood store, the odds of high (vs. low) intake of soft drink intake among adolescents increased by 21% (OR = 1.21).

A lower odd of high (vs. low) intake of soft drinks was found for every one-unit increase in the self-efficacy score, parental rules for soft drink intake, and perceived accessibility to the kiosk, although the associations were not statistically significant at the 0.05 level.

### Mediation analyses results

Results showed that adolescents with low-educated parents (OR = 2.12) and medium-educated parents (OR = 2.06) had higher odds of a high intake of soft drinks than their peers with high-educated parents.

Single mediation analyses results showed that perceived accessibility of SSB at home, parental modelling for SSB intake and frequency of food/drink purchased from the neighbourhood stores mediated the association between parental education and soft drink intake among adolescents.

Multiple mediation analysis results showed that perceived accessibility of SSB at home, parental modelling for SSB intake, and frequency of food/drink purchased from the neighbourhood stores were found to mediate the association between parental education and intake of soft drinks among adolescents. Among adolescents with low-educated parents, perceived accessibility of SSB at home explained 31.9% of the differences by parental education in adolescents’ soft drink intake compared to their peers with high-educated parents (OR = 1.20**)**. Similarly, among adolescents with a medium-educated parent, perceived accessibility of SSB at home explained 32.5% of the differences by parental education in adolescents’ soft drinks intake compared to their peers with high-educated parents (OR = 1.19). Among adolescents with a medium-educated parent, parental modelling for SSB intake explained 11.2% of the differences by parental education in adolescents’ soft drinks intake compared to their peers with high-educated parents (OR = 1.05**)**. Among adolescents with a low-educated parent, the frequency of food/drink purchased explained 15.8% of the differences by parental education in adolescents’ soft drink intake compared to their counterparts with high-educated parents (OR = 1.08**)**. After accounting for the mediators, the direct effect of parental education on adolescents’ soft drink intake was found to be significant for the low and medium parental education groups, indicating the association between parental education and soft drink intake among adolescents was partially mediated by the included mediators (Table 4).

**Table 4:** Multiple mediation analysis for the association between parental level of education and adolescents’ soft drinks intake (N* = 826)

	**a-path**		**b-path**	**c’-path**		**c-path**		Indirect effect (a*b)		**%Mediated**	
	**Parental education**			**Parental education**		**Parental education**		**Parental education**			
	Low	Medium		Low	Medium	Low	Medium	Low	Medium	Low	Medium
	B;95% CI	B; 95% CI	OR; 95%CI	OR;95% CI	OR; 95% CI	OR; 95% CI	OR;95% CI	OR 95% CI	OR95% CI		
Perceived accessibility of SSB at home	0.31 0.12, 0.49	0.32 0.17, 0.47	1.75 1.44, 2.15	1.74 1.12, 2.70	1.65 1.12, 2.45	2.12 1.43, 3.00	2.06 1.42, 3.18	**1.20** **1.09, 1.37**	**1.19** **1.09, 1.36**	31.9	32.5
Perceived parental modeling for SSB	0.14 -0.04, 0.34	0.18 0.03, 0.34	1.34 1.08, 1.67					1.04 0.99, 1.12	**1.05** 1.00*, 1.14	-	11.2
Frequency of food/drink purchase	0.46 0.18, 0.72	0.12 -0.07, 0.31	1.19 1.05, 1.36					**1.08** **1.02, 1.19**	1.02 0.99, 1.07	15.8	

Independent variable; parental education (reference; high), dependent variable (reference; low soft drink intake/week), c’-path represents the direct effect of parental education on soft drink intake (adjusted for the mediator), c-path represents the total effect of parental education on soft drink intake, bold values indicate statistically significant indirect effect. *OR* Odds ratio, *CI* confidence intervals, indirect effect with * represents variables significant at higher decimal values, *SSB* sugar-sweetened beverages

### Moderated mediation analysis results

Among the variables included in the moderated mediation model, a moderated mediation effect was observed for the perceived accessibility of SSB at home. No other moderated mediation effect was found. Accordingly, our results show that there is a significant moderation of the indirect effect of parental education on adolescents’ soft drink intake through perceived accessibility of SSB at home by neighbourhood-level income among adolescents with low-educated parents (index of moderated mediation (IMM), B = 0.25) and medium-educated parents (IMM, B = 0.22) compared to adolescents with higher-educated parents. We found a significant and relatively stronger conditional indirect effect of parental education on the intake of soft drinks among adolescents through perceived accessibility of SSB at home among adolescents with low-educated (OR = 1.31) and medium-educated (OR = 1.38) parents living in the low-neighbourhood income area. However, the conditional indirect effects of parental education on soft drink intake through perceived accessibility of SSB at home was weaker and non-significant for adolescents living in the high neighbourhood income area with an odds ratio of 1.02 and 1.11 for adolescents with low and medium-educated parents, respectively (Table 5).

**Table 5:** Conditional indirect effect of parental education on adolescents’ soft drinks intake through perceived accessibility of SSB at home by neighbourhood income (*N* = 826)

	**Low education (*n* = 213 (26.8%))**		**Medium education (*n* = 183 (22.2%))**	
	Low neighbourhood income	High neighbourhood income	Low neighbourhood income	High neighbourhood income
Conditional indirect effects OR (95% CI)	**1.31 (1.19, 1.56)**	1.02 (0.84, 1.26)	**1.38 (1.17, 1.72)**	1.11 (0.95, 1.29)
Index of moderated mediation effect B (95% CI)	**0.25 (0.003, 0.53)**		**0.22 (0.03, 0.48)**	

Independent variable; parental education (reference; high (*n* = 430, 52.1%), dependent variable; soft drinks intake (reference; lower intake /week), moderator variable; neighbourhood income (low vs high), mediator; perceived accessibility of SSB at home, *CI* bootstrapped confidence intervals, *OR* odds ratio, the model was adjusted for child sex, age, family structure, and data collection period, bold values statistically significant values, *SSB* sugar-sweetened beverages

## Discussion

Our results showed that increased parental modelling for SSB intake, higher perceived accessibility of SSB at home, and increased frequency of food/drink purchased from the neighbourhood stores were associated with a higher intake of soft drinks among adolescents. However, none of the neighbourhood food environment variables examined in this study was associated with adolescents’ intake of soft drinks. We also observed parental educational differences in the intake of soft drinks among adolescents and the observed differences were partially mediated by perceived parental modelling for SSB intake, perceived accessibility of SSB at home and frequency of food/drink purchased. Moderated mediation analysis showed that the mediating effect of perceived accessibility of SSB at home on the association between parental education and adolescent soft drink intake was stronger among those living in low-income neighbourhoods.

Increased parental modelling for SSB intake and higher perceived accessibility of SSB were associated with a higher intake of soft drinks among adolescents, as documented in previous studies which explored factors associated with SSB intake among adolescents [47–50]. These findings highlight the vital role of parents in influencing dietary behaviours among adolescents. Therefore, parents are an important group to target in interventions aimed at reducing the intake of soft drinks among adolescents. Our study indicated that higher frequency of food/drink purchased from the school/neighbourhood stores was associated with a higher intake of soft drinks among adolescents which is consistent with another study done in Norway [38]. Self-efficacy is a strong determinant of health behaviours which has been used as a construct for theories such as the social cognitive theory [51]. Previous studies have shown an inverse association between self-efficacy for avoiding SSB or for healthy eating and SSB intake among adolescents [52, 53]. However, in the present study, the association between self-efficacy and adolescents’ soft drink intake was not significant.

Our results found parental education differences in soft drink intake among adolescents, which is in line with other studies indicating socioeconomic differences in SSB [6, 7, 11, 12]. The parental education differences in adolescents’ soft drink intake in our study were explained by parental modelling for SSB intake, perceived accessibility of SSB at home and frequency of food/drink purchased. The mediators, parental modelling and accessibility of soft drinks at home identified in this study were reported as consistent mediators in a previous systematic review study [29]. Thus, targeting these mediators for public health interventions could help tackle socioeconomic differences in the intake of soft drinks with added sugar among adolescents.

Moderated mediation analysis results showed that the mediating effect of perceived accessibility of SSB at home on the association between parental education and adolescent soft drink intake was stronger among those living in low neighbourhood income areas.

These differences could be due to the presence of social norms in the neighbourhoods that could influence healthy or unhealthy dietary behaviours among adolescents. In this regard, a qualitative study from Oslo found that adolescents living in a higher neighbourhood income area had social norms facilitating healthy dietary behaviours through a limited serving of unhealthy food options at home [54]. On the other hand, an absence of shared social norms favouring healthy dietary behaviour was a barrier to healthy eating among adolescents in lower-income areas [54]. Thus, public health interventions ensuring access to healthy foods, especially for those adolescents living in low-income neighbourhood areas, are essential to reduce socioeconomic differences in the intake of soft drinks among adolescents.

Our study shows that none of the factors of the neighbourhood food environment (both perceived and objectively measured) mediates parental education differences in adolescent soft drink intake. However, evidence of parental education differences in the perceived (i.e. perceived travel time to the nearest fast-food shops, perceived neighbourhood accessibility of fast-food shops) and objectively measured (i.e. distance to the nearest grocery store and density of grocery stores, fast-food outlets and restaurants) neighbourhood food environment was found. Nonetheless, none of the neighbourhood-level factors that differed by parental education in our study was associated with adolescents’ soft drink intake. Our findings were consistent with two North American studies [55, 56]. However, our results were not in line with another study from the USA, which found associations between SSB intake with distance-based measures of food environments (i.e. distance from home to the nearest restaurant and grocery stores) and density of food retails (i.e. restaurant of any kind, convenience store, fast-food restaurant, grocery store or any retail facility) [57]. The statistically non-significant association of the neighbourhood food environment factors with adolescents’ soft drink intake may be partly because adolescents included in our study (i.e. mean age of 12.4 years) have limited autonomy to go out for food or purchase food from the neighbourhood food retailers. Nevertheless, the neighbourhood environment may have more influence on soft drink intake as the adolescents grow older, when they acquire more autonomy to go out for food or purchase food from neighbourhood food retailers [58]. 

### Implications for practice and research

Our results found important contributions of modifiable factors at the intrapersonal and interpersonal levels namely parental modelling for soft drink intake; perceived accessibility of soft drinks at home and frequency of food/drink purchased from neighbourhood shops to a higher intake of soft drinks and explaining parental education differences in intake among 12-year-old adolescents in Oslo, Norway. Parental modelling of soft drink intake and perceived accessibility of soft drinks at home were also shown to be consistent mediators by a systematic review study which explored mediators of socioeconomic differences in dietary behaviours including soft drink intake among youth in high-income countries [29]. Thus, health promotion efforts aimed at promoting healthy eating behaviours and limiting home accessibility of soft drinks can be considered whenever feasible. In addition, perceived accessibility of soft drinks at home was found to be an important contributor to the parental education differences in adolescents' soft drink intake among those living in low neighbourhood-income areas. Thus, public health efforts aimed at limiting access to soft drinks among residents in low-income neighbourhoods may be particularly important to reduce parental education differences in adolescents' soft drink intake in similar settings. Limiting the frequency of food/drink purchased by adolescents from neighbourhood shops could be considered to reduce adolescents’ soft drink intake and the differences in the intake by parental education. In this regard, Norway had introduced taxes on confectionary and non-alcoholic beverages [59], students have limited access to neighbourhood stores during school hours, and currently, the government agreed to implement a new law requiring age restrictions (16 years) for buying energy drinks.

Targeting these intrapersonal and interpersonal level factors alone may not be sufficient to reduce socioeconomic differences in soft drinks intake among adolescents, given that health behaviours such as soft drink intake result from an interaction of factors at multiple levels [60]. In this regard, the individual’s food preferences (e.g. healthy food preferences) may be influenced by the neighbourhood availability and accessibility of healthy food options. Similarly, the availability and accessibility of healthy food options at home can be affected by broader-level factors such as the neighbourhood availability and accessibility of healthy food options, price and market policies. In addition, evidence of an increase in inequalities between socioeconomic groups for downstream interventions (e.g. interventions targeting individual-level factors) and a decrease in the inequalities for upstream interventions (e.g. interventions on social or policy level determinants) has been documented [61]. Thus, knowledge of both factors at the lower levels (e.g. intrapersonal and interpersonal levels) and broader levels are important to design effective strategies to tackle socioeconomic differences in soft drink intake among adolescents.

### Strengths and weakness of the study

Our study has several strengths. This study utilised both perceived and objective measures of neighbourhood food environment exposures and explored correlates of adolescents’ soft drink intake at different levels. Our study provides new information regarding the moderating roles of neighbourhood income for the indirect effects of parental education on adolescents’ soft drink intake.

We have used a cross-sectional study design. This could be a limitation given that cross-sectional data cannot allow inference about causality, and mediation analysis should ideally be performed using longitudinal data. An overrepresentation of parents with high education is another limitation of this study. The intake of non-carbonated soft drink was not included as an outcome in this study. Thus, the total consumption of SSBs has likely been underestimated. Future studies should include other sugar-sweetened beverages such as cordials and energy drinks.

## Conclusions

Our study identified several modifiable factors at the intrapersonal and interpersonal levels (parental modelling for SSB intake; perceived accessibility of SSB at home and frequency of food/drink purchased from neighbourhood shops) which could be targeted to reduce soft drink intake and related parental education differences among adolescents. Perceived accessibility of SSB at home was shown to be an important contributor to the parental education differences in adolescents' soft drink intake among those living in low neighbourhood income areas. Thus, public health efforts limiting access to soft drinks among residents in low-income neighbourhoods may be particularly important to reduce parental education differences in adolescents' soft drink intake in similar settings.

## Data Availability

The dataset used in this study is not yet publicly available because of ongoing data analyses.
